# Altered asymmetries of the structural networks comprising the fronto-limbic brain circuitry of preterm infants

**DOI:** 10.1038/s41598-020-79446-0

**Published:** 2021-01-14

**Authors:** Joo Young Lee, Yong-Ho Choi, Jong Ho Cha, Ji Young Lee, Young-Jun Lee, Bo-Hyun Kim, Il-Kewon Kim, Jong-Min Lee, Hyun Ju Lee

**Affiliations:** 1 Department of Pediatrics, Hanyang University Hospital, Hanyang University College of Medicine, 17 Haengdang-dong, Seongdong-gu, Seoul, 133-792 Republic of Korea; 2 Department of Biomedical Engineering, Hanyang University, Seoul, Republic of Korea; 3 Department of Radiology, Hanyang Hospital, Hanyang University College of Medicine, Seoul, Republic of Korea; 4 Division of Neonatology and Developmental Medicine, Seoul Hanyang University Hospital, Seoul, Republic of Korea

**Keywords:** Health sciences, Medical research

## Abstract

This study aimed to elaborate upon prior findings suggestive of the altered lateralization of structural connectivity in the developing preterm brain by using diffusion tensor imaging tractography to explore how network topological asymmetries in fronto-limbic neural circuitry are altered at 36–41 weeks, postmenstrual age in 64 preterm infants without severe brain injury and 33 term-born infants. We compared the pattern of structural connectivity and network lateralization of the betweenness centrality in the medial fronto-orbital gyrus, superior temporal gyrus, amygdala, and hippocampus—the structures comprising the fronto-limbic brain circuit—between preterm and term infants. Global efficiency, local efficiency, and small-world characteristics did not differ significantly between the two hemispheres in term-born infants, suggesting that integration and segregation are balanced between the left and right hemispheres. However, the preterm brain showed significantly greater leftward lateralization of small-worldness (*P* = 0.033); the lateralization index of the betweenness centrality revealed that the medial fronto-orbital gyrus (*P* = 0.008), superior temporal gyrus (*P* = 0.031), and hippocampus (*P* = 0.028) showed significantly increased leftward asymmetry in preterm infants relative to term-infants independent of sex, age at imaging, and bronchopulmonary dysplasia. The altered lateralization of fronto-limbic brain circuitry might be involved in the early development of social–emotional disorders in preterm infants.

## Introduction

Significant recent improvements in the capacity of the neonatal intensive care unit to administer preterm care have engendered a burgeoning interest in the neurodevelopment of premature infants. Accumulating evidence indicates that such children suffer from multiple neurodevelopmental disorders; indeed, this population features 75% and 7% prevalence of developmental disabilities and autism spectrum disorders (ASD), respectively^1–3^. Moreover, why some preterm birth induces adverse long-term neurodevelopmental effects despite exhibiting no measurable focal brain injury on magnetic resonance imaging (MRI) remains unclear.

Recent studies using advanced neuroimaging techniques have revealed meaningful differences in white matter microstructure and structural brain networks between preterm infants and term-born neonates. Specifically, preterm infants were observed to exhibit aberrant structural connectivity, including global efficiency, local efficiency, and small-worldness, in major tracts connecting the corticospinal tracts, commissural fibres, and cerebellum^4^. However, while we have previously shown that preterm infants exhibit significantly lower global and local efficiency, increases in small-worldness indicate an enhanced resilience against prematurity-related pathology^5^.

Cerebral lateralization refers to the functional specialization of the two cerebral hemispheres: e.g., language production and motor functions are lateralized to the left cerebral hemisphere and emotional processing to the right. This spatial segregation of dynamic brain networks is reportedly a critical process in early neurodevelopment^6^, the impairment of which is associated with premature birth even in the absence of focal brain injury^7^. While Kwon et al.^8^ demonstrated that preterm neonates show a significant lack of lateralization in regions involving both receptive and expressive language, the mechanisms mediating social–emotional processing remain poorly characterized.

The aberrant network connectivity of the fronto-limbic circuit has been implicated in emotion, social communication, psychiatric, and neurodevelopmental disorders^9,10^. A previous study of 6-month-old healthy infants demonstrated that the rightward lateralization of the fronto-limbic brain development of white matter microstructure helps to support the development of social communication skills^11^. Other research performed across the past decade suggests that altered lateralization of the neural networks comprising the fronto-limbic circuit contributes to social and emotional problems in children and adults^12–14^. To date, however, no study has investigated cerebral lateralization in the fronto-limbic connection of the structural brain networks of preterm neonates. To address this dearth in the literature, we studied the asymmetry of both global and local networks of the fronto-limbic circuit in the neonatal brain using diffusion tensor imaging (DTI) tractography and structural network analysis. We hypothesized that preterm birth results in architectural changes in the global network or specific fronto-limbic connections at near-term age. Furthermore, we speculated that the altered lateralization of preterm brain in fronto-limbic connection might be related to social–emotional scores at 18 months of corrected age in the developing preterm brain.

## Results

Table 1 presents the prenatal and neonatal data of both groups. Compared to the full-term infants, preterm infants had a significantly lower gestational age (30.08 ± 3.94 vs. 38.39 ± 1.22, *P* < 0.001) and lower birth weight (1423.61 ± 588.44 vs. 3202.63 ± 326.40, *P* < 0.001). Preterm infants exhibited significantly lower 1-min and 5-min Apgar scores. With respect to comorbidities, grade 1 intraventricular haemorrhages (*P* = 0.054), culture-proven sepsis (*P* = 0.102), and bronchopulmonary dysplasia (*P* < 0.001) were more frequent among preterm infants. Neurodevelopmental outcome data were available at 18 months of age (corrected for the prematurity) for 57 out of 64 (89%) of the preterm infants and for 21 out of 33 (64%) of the term infants. The preterm infants had significantly lower mean composite scores on cognitive (95.47 ± 14.47 vs. 102.38 ± 8.74, *P* = 0.013), motor (95.38 ± 16.41 vs. 105.04 ± 11.03, *P* = 0.015) and social–emotional (97.28 ± 17.39 vs. 108.57 ± 11.95, *P* = 0.008) domains compared with term-born infants. The incidence of language delay was significantly higher among preterm infants than term infants (42.1% vs. 10%). Thirteen (13/57, 22.8%) preterm infants had a Bayley-SE score of < 85—i.e., > 1 SD below the standardized mean for the assessment. No term infant had a Bayley-SE score of < 85.

**Table 1:** Demographic, clinical characteristics and neurodevelopmental outcome of preterm infants and term infants.

Variables	Preterm (n = 64)	Term (n = 33)	*P* value
**Perinatal characteristics**			
Caesarean section	54/64 (84.4)	9/33 (27.2)	0.001
Antenatal corticosteroids	29/64 (45.3)	0	< 0.001
Preeclampsia	8/64 (12.5)	0	0.102
GDM	5/64 (7.8)	2 (6.1)	0.669
Histologic chorioamnionitis	30/64 (51.5)	-	NA
**Infant characteristics**			
Gestational age, wk	30.08 ± 3.94	38.39 ± 1.22	< 0.001
Birth weight, g	1423.61 ± 588.44	3202.63 ± 326.40	< 0.001
Male sex	13/64 (39.4)	8/33 (34.8)	0.785
Apgar 1 min	4.02 ± 1.69	6.73 ± 2.12	< 0.001
Apgar 5 min	6.58 ± 1.31	8.40 ± 1.35	< 0.001
PDA	30/64 (46.9)	0	< 0.001
IVH, grade 1	11/64 (17.2)	1 (3.0)	0.054
Culture-proven sepsis	8/64 (12.5)	0	0.102
NEC (requiring surgery)	3/64 (4.7)	0	0.315
ROP (requiring surgery)	6/64 (9.4)	0	0.092
Days on mechanical ventilator	14.26 ± 20.79	3.23 ± 1.25	0.985
Bronchopulmonary dysplasia	29/64 (45.3)	0	< 0.001
Age at MRI scan, wk	37.30 ± 1.33	38.61 ± 0.86	0.171
**Maternal characteristics**			
Maternal age, years	33.38 ± 4.420	33.35 ± 4.33	0.982
Maternal smoker	1 (1.5)	0	1.000
Maternal education			0.379
Less than high school	14 (21.9)	5 (15.2)	
High school graduate	49 (76.6)	26 (78.8)	
College/university graduate	1 (1.6)	2 (6.1)	
**Neurodevelopmental follow-up at 18 mo of age**			
Cognitive composite score, mean (SD)	95.47 ± 14.47 (57/64)	102.38 ± 8.74 (21/33)	0.013
Infants with scores < 85, n (%)	10/57 (17.5)	0 (0)	0.056
Language composite score, mean (SD)	89.21 ± 15.42 (57/64)	93.95 ± 6.68 (21/33)	0.063
Infants with scores < 85, n (%)	24/57 (42.1)	2 (10)	0.012
Motor composite score, mean (SD)	95.38 ± 16.41 (57/64)	105.04 ± 11.03 (21/33)	0.015
Infants with scores < 85, n (%)	11/57 (19.3)	0 (0)	0.057
S–E composite score, mean (SD)	97.28 ± 17.39 (57/64)	108.57 ± 11.95 (21/33)	0.008
Infants with scores < 85, n (%)	13/57 (22.8)	0 (0)	0.017

Data are presented as the mean ± SD or number (%).

*GDM* gestational diabetes mellitus, *PDA* patent ductus arteriosus, *IVH* intraventricular, *NEC* necrotizing enterocolitis, *ROP* retinopathy of prematurity, *MRI* magnetic resonance imaging, *S–E* social–emotional.

We compared the differences in local efficiency, clustering coefficient, global efficiency, path length, and the small-worldness between preterm infants and term-born neonates. These measures were comparable between the two groups (Table 2; all *P* > 0.05). The small worldness tended to be higher in preterm infants than term-born infants. We quantified the betweenness centrality for the fronto-limbic connections: the medial fronto-orbital gyrus (mFOG), superior temporal gyrus (STG), amygdala, and hippocampus. However, the betweenness centrality of the mFOG (0.019 ± 0.024 vs. 0.010 ± 0.014, *P* = 0.169), STG (0.048 ± 0.042 vs. 0.046 ± 0.042, *P* = 0.599), and hippocampus (0.007 ± 0.009 vs. 0.005 ± 0.007, *P* = 0.082) in the left hemisphere tended to be higher in preterm infants than in term-born infants independent of age at MRI and bronchopulmonary dysplasia.

**Table 2:** Network measures of the left and right hemisphere in the preterm and term infants.

Variables	Left			Right		
	Preterm (n = 64)	Term (n = 33)	*P* value*	Preterm (n = 64)	Term (n = 33)	*P* value*
**Global brain network**						
Local efficiency	0.778 ± 0.036	0.762 ± 0.049	0.425	0.790 ± 0.042	0.775 ± 0.051	0.587
Global efficiency	0.674 ± 0.029	0.673 ± 0.026	0.519	0.682 ± 0.032	0.684 ± 0.028	0.081
Path length	1.694 ± 0.078	1.704 ± 0.083	0.523	1.675 ± 0.095	1.675 ± 0.084	0.113
Small-worldness	1.219 ± 0.116	1.191 ± 0.107	0.623	1.179 ± 0.108	1.180 ± 0.105	0.717
**Betweenness centrality of FLC**						
mFOG	0.019 ± 0.024	0.010 ± 0.014	0.169	0.006 ± 0.008	0.007 ± 0.011	0.079
STG	0.048 ± 0.042	0.046 ± 0.042	0.599	0.039 ± 0.023	0.047 ± 0.036	0.830
Amygdala	0.001 ± 0.005	0.001 ± 0.001	0.107	0.001 ± 0.002	0.001 ± 0.001	0.570
Hippocampus	0.007 ± 0.009	0.005 ± 0.007	0.082	0.004 ± 0.004	0.008 ± 0.019	0.155

Data are presented as the mean ± SD.

*FLC* fronto-limbic circuit, *mFOG* medial fronto-orbital gyrus, *STG* superior temporal gyrus.

*Adjusted for sex, age at MRI scan and bronchopulmonary dysplasia.

**Figure 1:**
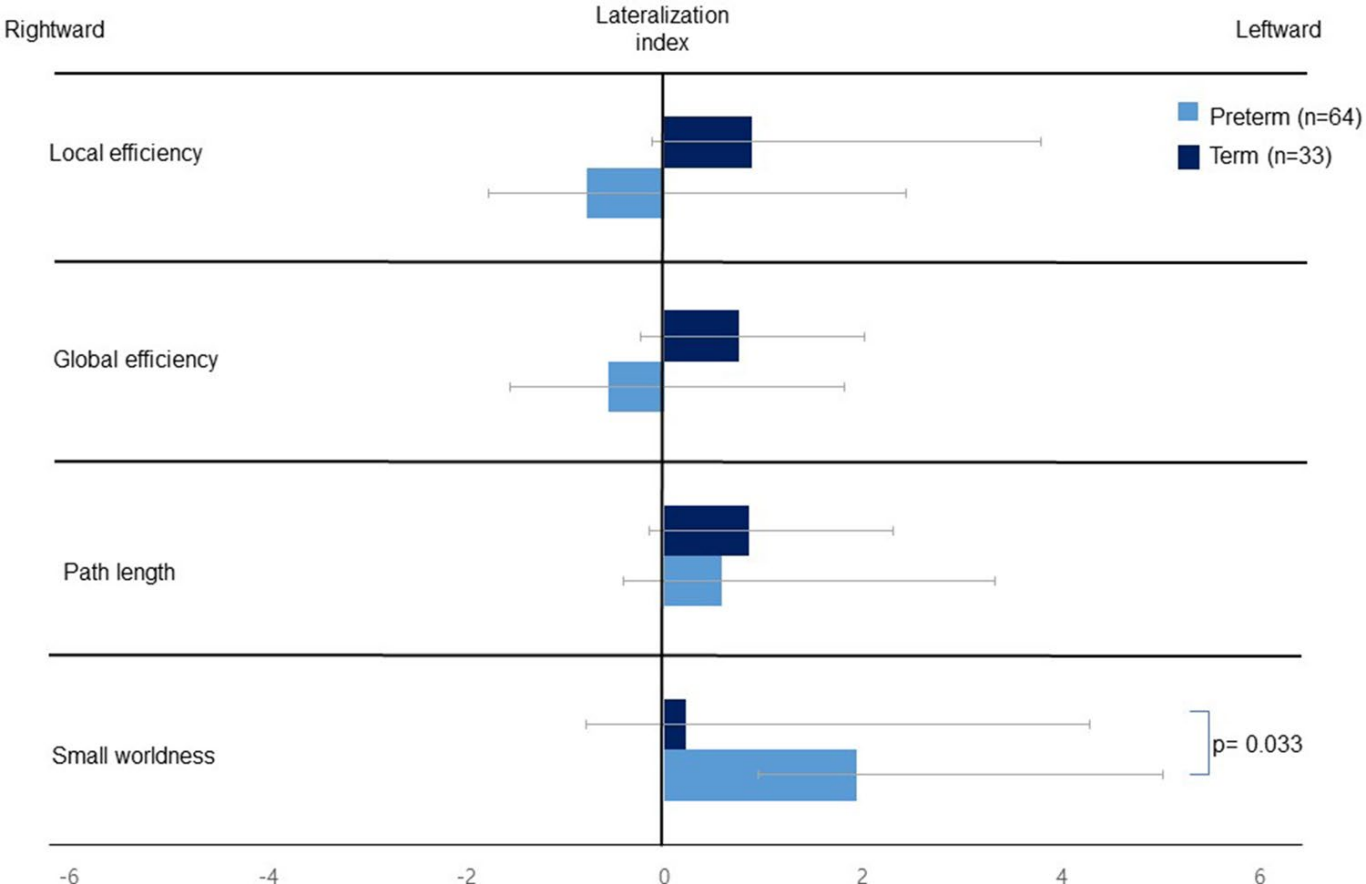
The comparison of the lateralization index of the global network metrics between preterm and full-term infants. We controlled for sex, age at MRI scan, and bronchopulmonary dysplasia when performing the analyses. *P* values of ≤ 0.05 were considered to indicate statistical significance. All *P* values were corrected for the false-discovery rate.

The results of the statistical analysis of the lateralization index of the global network metrics are summarized in Fig. 1. While the left hemisphere tended to be more efficient with respect to global and local efficiency in term-born infants, the right hemispheric predirection of global and local efficiency was observed in preterm infants. However, a left-hemispheric predominance in the small-world properties was observed in both groups. When comparing the lateralization index of the small-worldness between the 2 groups, preterm infants exhibited significantly more leftward lateralization than did the full-term group, suggesting the prominence of small-world properties in the left hemispheres of preterm infants (1.942 ± 4.488 and 0.212 ± 4.076, *P* = 0.033).

**Figure 2:**
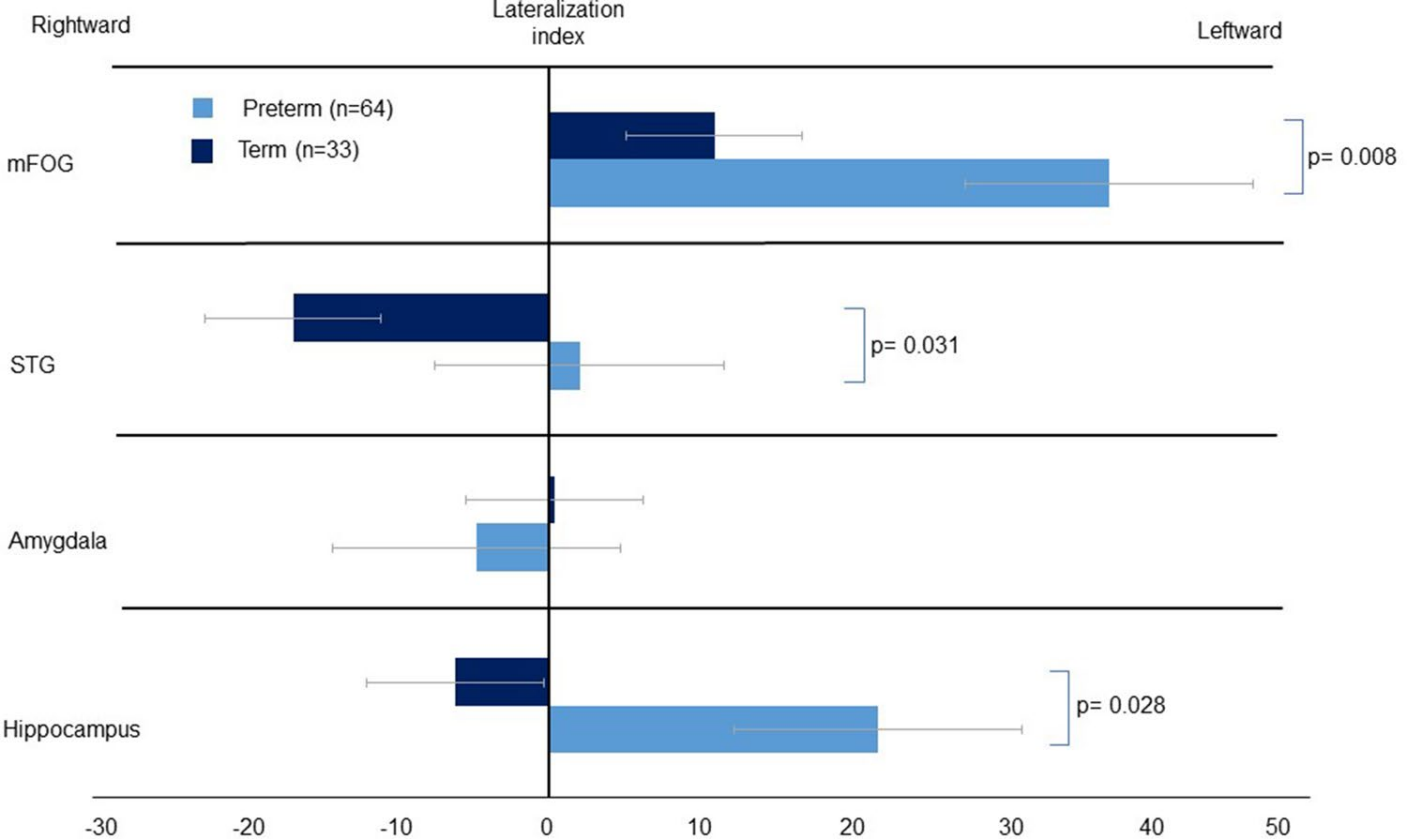
The comparison of the lateralization index of the betweenness centrality in the fronto-limbic network of both preterm and full-term infants. We controlled for sex, age at MRI scan, and bronchopulmonary dysplasia when performing the analyses. *P* values of ≤ 0.05 were considered to indicate statistical significance. All *P* values were corrected for the false-discovery rate. Abbreviations: mFOG, medial froto-orbital gyrus; STG, superior temporal gyrus.

Figure 2 presents the comparison of the betweenness centrality in the fronto-limbic network of both preterm and full-term infants; in the latter group, the mFOG was associated with a leftward asymmetry of betweenness centrality, while the STG and hippocampus were associated with a rightward asymmetry. However, the mFOG of preterm infants exhibited a significantly greater leftward asymmetry than did their full-term counterparts (36.823 ± 26.562 vs. 10.855 ± 28.167, *P* = 0.008). The rightward predominance of the betweenness centrality in the STG and hippocampus of term-born controls was not observed in preterm infants. When comparing the lateralization index of the fronto-limbic cortex between preterm and full-term infants, the mFOG (36.823 ± 26.562 vs. 10.855 ± 28.167, *P* = 0.008), STG (2.001 ± 25.551 vs. − 16.820 ± 21.777, *P* = 0.031), and hippocampus (21.636 ± 30.623 vs. − 6.167 ± 32.312, *P* = 0.028) of preterm infants was observed to exhibit significantly greater leftward asymmetry after correcting for sex, age at MRI, and bronchopulmonary dysplasia.

**Figure 3:**
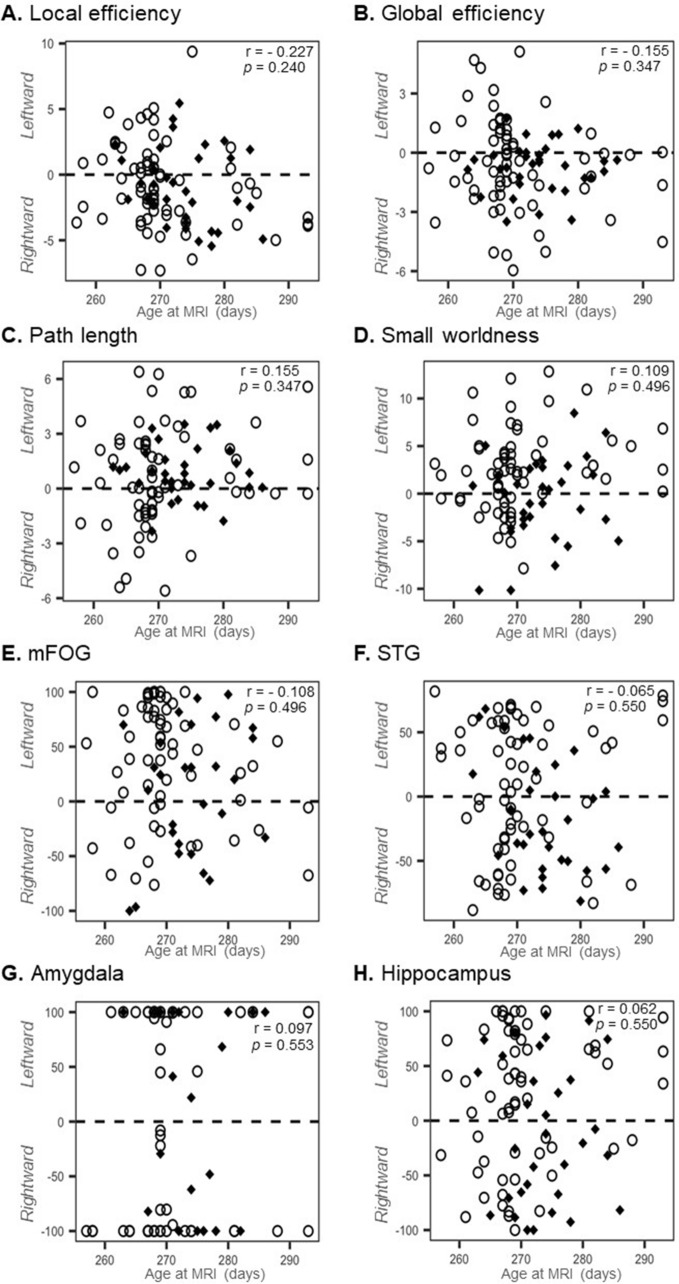
The features of global network asymmetries and fronto-limbic asymmetry were plotted against age at which all infants underwent the MRI scan (n = 97). *P* values of ≤ 0.05 were considered to indicate statistical significance. All *P* values were corrected for the false-discovery rate. Abbreviations: mFOG, medial fronto-orbital gyrus; STG, superior temporal gyrus.

**Figure 4:**
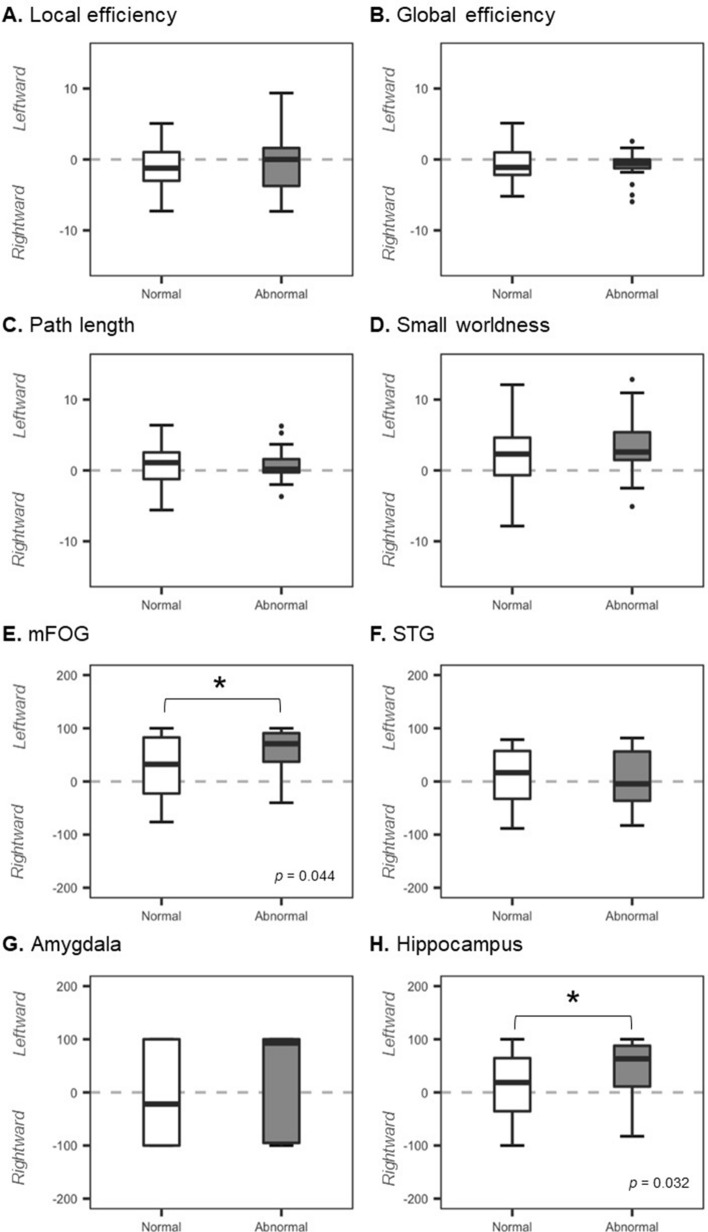
Comparison of network topological asymmetries between 57 preterm infants with normal scores on the Bayley-III socio-emotional scale (≥ 1SD below average, n = 44) and abnormal scores on the socio-emotional scale (< 1SD below average, n = 13). We controlled for gestational age and age at MRI scan when performing the analyses. An asterisk indicates statistical significance. *P* values of ≤ 0.05 were considered to indicate statistical significance. All *P* values were corrected for the false-discovery rate. Abbreviations: mFOG, medial fronto-orbital gyrus; STG, superior temporal gyrus.

Our assessment of how the age at MRI scan affects the asymmetry of the global network, mFOG, STG, amygdala and hippocampus at 36–41 weeks of corrected age revealed no correlation between age at MRI scan and global network or fronto-limbic asymmetries in all infants (n = 97) (Fig. 3). Figure 4 shows the subgroup analyses for the comparison of network topological asymmetries between 57 preterm infants with abnormal Bayley-SE (< 1SD below average, n = 13) and normal Bayley-SE (≥ 1SD below average, n = 44). The preterm infants with abnormal Bayley-SE exhibited greater leftward lateralization of global and local efficiency and small-world properties than did those with normal Bayley-SE. After controlling for gestational age and age at imaging, we found significant differences in the leftward asymmetry of the betweenness centrality in the mFOG (*P* = 0.044) and hippocampus (*P* = 0.032) between preterm infant with normal and abnormal Bayley-SE.

## Discussion

The present study observed fronto-limbic asymmetry in the structural brain networks of preterm infants by using DTI and structural network analyses. The preterm infants with no evidence of severe brain injury exhibited significantly more leftward lateralization of small-world properties than did their full-term counterparts. Moreover, we found the STG and hippocampi of preterm infants to feature significantly greater leftward asymmetries in betweenness centrality, and their mFOG to exhibit greater activation, than those of full-term infants.

DTI has revealed that the hemispheric structural asymmetry in the brains of children involves not only regional brain volumes but also white matter microstructure^15,16^. Despite evidence indicating a variety of regional differences in the hemispheric asymmetry of term-born children, patterns of neural network asymmetry in preterm infants still require elucidation. Ratnarajah et al.^17^ recently reported structural connectivity asymmetry in 124 full-term neonates, indicating specific lateralized brain functions in the topology of each cerebral hemisphere. Moreover, leftward asymmetric efficiency at both global and local levels was found in the term-born brain, which may reflect the early development of the lateralization of neurological functions at birth. While this finding was consistent with our observation that full-term infants exhibit the leftward lateralization of global and local efficiency, we further found that preterm infants exhibit the rightward lateralization of global and local efficiency and a more balanced information propagation with marked small-worldness in the left cerebral hemisphere relative to full-term infants. These findings indicate the possible compensatory development of neural networks in preterm infants and suggest that the adaptive growth of preterm brain networks was preserved and enhanced following the stabilization of their clinical status. Recent investigations into the small-world property have significantly impacted the understanding of the topological organization of the neonatal brain network in early life^18^. Furthermore, the present results agree with our previous observation that structural brain networks after preterm birth are reorganized to accelerate the development of small-world property in preterm infants without apparent brain abnormalities at term-equivalent ages^5^.

Despite considerable advances in the survival rate among preterm infants, the mechanisms underlying the development of neural asymmetry present at or before birth—especially those concerning the alteration of the lateralization of structural connectivity—have lacked rigorous exploration. The few existing studies on full-term neonates have focused on the leftward predominance of language and motor functions and the larger gray and white matter volumes at the areas associated with these functions, as well as early white matter maturation in the left hemisphere^16,19^. A recent neuroimaging study of 26 very preterm infants and 25 controls revealed that cerebral lateralization in the left language region, including Wernicke's and Broca’s areas, was impaired in the preterm group at term-equivalent ages^8^.

Our study demonstrated that social–emotional development at 18 months of age, as assessed by the maternal completion of the Bayley-SE Scale, was diminished in preterm infants relative to term-born neonates. However, since subtle impairments may not be evident in infancy and can either improve or worsen during preschool age, evaluations performed at a corrected age of 18 months should be further explored by follow-up studies to consolidate the present results. This possibility was anticipated by early findings that preterm infants of less than 27 weeks of age were three times more likely to develop ASD than controls after controlling for sex, SGA, and maternal education^20^. The study^20^ further suggested that preterm infants of < 34 weeks of age who were observed in the neonatal intensive care unit to have potential risk factors such as low-grade intraventricular haemorrhage or high-frequency ventilation were likely to have a relatively high risk of developing ASD. Notably, the differential activation of the right hemispheric limbic structures is believed to be determined and established during prenatal and postnatal development^17^, indicating that the right hemisphere of the neonatal brain has its own dense, efficient network for social and emotional processing. Ratnarajah et al.^17^ suggested that limbic structures, such as the cingulate gyrus and hippocampus, of term-born infants show a rightward asymmetry in the betweenness centrality of emotional processing, suggesting that brain regions involved in emotional processes play crucial roles in efficient communication in the right hemisphere. However, few studies have considered the correlation between the pathophysiology of socioemotional processing and social–emotional delay among preterm infants using DTI-based connectomes.

Frontotemporolimbic connections are important interactive pathways involving the “social brain” via connections between the mFOG, STG, amygdala, and hippocampus. This network lateralization to the right hemisphere at the neonatal stage could be associated with social information processing and emotional regulation in later neurodevelopment. The presently observed rightward asymmetry of betweenness centrality of the STG and hippocampus in term-born controls is consistent with previous findings^17,21^. Altered lateralization in these regions favoring the left hemisphere rather than the right might contribute substantially to the early onset of social–emotional disorders in preterm infants^9^.

Our results suggest that the leftward lateralization of the STG and hippocampus in preterm infants may contribute to the maladaptive development of social communication by altering the network connectivity of the fronto-limbic circuit and thus compromising normative social–emotional processing. This conclusion is partially supported by research on the asymmetry of FA values of the uncinate fasciculus: the largest white matter association tract connecting the prefrontal cortex, superior temporal cortex, and limbic system^11^. Specifically, the right uncinated fasciculus connecting the mFOG, amygdala, and STG at 6 months was found to be a strong predictor of the emergence of joint attention at 9 months of age^11^. The asymmetry of the mFOG and STG is considered to be associated with a rightward shift according to the infant’s maturation pattern, which should be lateralized to the right at term-equivalent ages in neonates^11,22^. However, we observed an excessive leftward asymmetry that involved redundant leftward lateralization of the mFOG in preterm infants relative to their full-term counterparts; this finding agrees with previous observations on functional MRI of reduced activation in the right mFOG of patients with ASD relative to controls^23^.

To date, no study has reported significant differences in the lateralization of the fronto-limbic circuit between preterm and term-born infants. This study is, to the best of our knowledge, the first to identify the altered lateralization of betweenness centrality in the efficiency of the networks of the fronto-limbic circuit with DTI performed at the term-equivalent age in preterm infants. We further addressed these connections in relation to the Bayley-SE scale scores of infants without brain injuries at 18 month of corrected age. However, the present study is subject to several limitations: (1) The relatively small sample size in term-born neonates may have resulted in insufficient power to detect possible associations. This is partially due to the high cost of data collection and the substantial lack of term-born neonates for follow-up. (2) The confirmation of an association between socio-emotional development and fronto-limbic asymmetry was limited by our small number of preterm infants with low socio-emotional scores and by the preterm infants not having been able to undergo more assessments for the subdomain of behavior at older ages. (3) We analyzed the development of the central nervous system in children at an age when it is undergoing constant change, and its correlation with clinical development is highly dynamic. Hence, the development or alterations of the nervous systems of the included preterm infants may have been delayed at the point of observation—despite our having collected data at a corrected age of 18 months after birth. The value of the correlative analysis between anatomical and functional findings should be assessed by larger prospective cohort studies.

The present study benefitted from recruited a large number of preterm infants with available 3 T MRI imaging data at near-term age, allowing for the exploration of distinct white matter tracts, rather than the WM microstructure in its entirety, in relation to socio-emotional development in children born preterm. We focused on infants without significant brain injuries, using network topological asymmetries in fronto-limbic neural circuitry. Our results may implicate the alteration of fronto-limbic brain circuitry in the early development of social–emotional disorders in preterm infants. The present study further indicates that the altered interhemispheric leftward asymmetries of small-worldness in the global network and fronto-temporo-limbic areas may help to elucidate the etiology of neurodevelopmental disorders across the long-term follow-up of preterm infants.

## Methods

The present study recruited 83 preterm infants from the Hanyang Inclusive Clinic for Developmental Disorders, Hanyang University Seoul Hospital, between December 2016 and April 2018. Our sample included infants with no evidence of genetic disorders, congenital infections, or congenital brain abnormalities, and for whom MRI data obtained at near-term age (GA, 36–41 weeks) was available. We excluded eight infants with (1) intraventricular or intracranial hemorrhage greater than grade I on brain ultrasound at 3 days, 7 days, or 3 weeks of birth; (2) periventricular leukomalacia or white matter injury on term-equivalent MRI; or (3) intrauterine growth retardation. Of the 83 eligible preterm children, 75 met our inclusion criteria. 33 term-born infants (GA range, 37–42 weeks) with normal MRI findings and neurodevelopment were also recruited for the term-born control group. Eleven of the remaining 75 preterm infants were removed due to poor image quality that precluded DTI processing; hence, the initial sample included 64 datasets. Our final sample of preterm infants and term-born infants included 64 and 33 datasets, respectively. Images were obtained from the controls and preterm infants during natural sleep without sedation. The infants underwent follow-up neurosensory and neurodevelopmental assessments at 4, 9, and 18 months of corrected age. Demographic data included maternal details, GA, birth weight, sex, and Apgar scores. Neonatal outcomes included patent ductus arteriosus, culture-proven sepsis, necrotizing enterocolitis, retinopathy of prematurity, and bronchopulmonary dysplasia. This present study protocol and scanning procedure were approved by the institutional review board of Hanyang University Hospital (IRB No. 2017-04-004-002). All procedures were performed in accordance with the principles of the Declaration of Helsinki, and informed consent for participation was obtained from the children's parents prior to their participation in the study.

### Neurodevelopmental assessment

The Bayley Scales of Infant and Toddler Development, Third Edition (BSID-III)^24^, were used to perform the neurological and developmental assessments at Hanyang Inclusive Clinic for Developmental Disorders when the children were 18 months of age (corrected for prematurity). The routine neurological examination was completed by a developmental pediatrician, while the developmental assessment was performed by paediatric physiotherapist and neonatologist. Seven of the preterm infants (11%) did not complete the Bayley-III test at 18 months of corrected age; the parents of three of the infants did not permit the assessment of their children; two infants dropped out of the study during follow-up, and two of the participants were withdrawn from the study because of their inability to cooperate with testing.

Developmental outcomes included cognitive, language, motor, and social–emotional composite BSID-III scores based on the child’s adjusted age at the time of evaluation. Assessments of the cognitive, language, and motor domains were performed using the items administered to the child, while assessments of social–emotional domains were informed by the primary caregiver’s responses to a questionnaire. The normative means (standard deviation [SD]) of each outcome score was 100; scores of less than 85 (1 SD below average) were considered to indicate neurodevelopmental delay.

### MRI

All scans were performed on 3 T MRI (Philips Real-Time Compact Magnet, Achieva 3.0-T Xseries, 16-channel SENSE head coil). The T1-weighted images included sagittal and axial T1 spin-echo sequences (400/25/2, repetition time [TR]/echo time [TE]/signal intensity averages) and axial T2 Turbo spin-echo sequences (3000/100/1). DTIs were obtained using a single-shot spin-echo planar sequence with a SENSE factor of two and an echo-planar imaging factor (TR/TE, 5243/76 ms; voxel size, 1.97 × 1.97 × 2 mm; field-of-view, 150 mm, 45 axial sections). Diffusivities were measured along 15 directions with an electrostatic gradient model (b = 800). No sedation was used and the infants remained asleep in the scanner by feeding them before the scan. A pulse oximeter was monitored to ensure stable heart rate and respiration.

### Image processing

The diffusion-weighted images were processed using the FMRIB Software Library (http://www.fmrib.ox.ac.uk/fsl in the public domain). Motion artifacts and eddy current distortions were corrected by normalizing each diffusion-weighted volume to the nondiffusion-weighted volume (b0) with the FMRIB Linear Image Registration Tool. Each brain mask was created using the Brain Extraction Tool (BET). Subsequently, DTIs were reconstructed for each voxel using DTIFit in FMRIB’s Diffusion Toolbox. Fractional anisotropy (FA) and mean diffusivity (MD) values were then calculated. Whole-brain deterministic diffusion fiber tracking was performed for each infant using quantitative anisotropy in DSI Studio (dsi-studio.labsolver.org) and was estimated using a streamlined algorithm in DSI Studio^25^. Fiber tracking was initiated from the center of each voxel and proceeded to adjacent voxels by following the direction of water diffusivity. Fiber tracking was stopped when the FA value fell below 0.1 and if the streamline turned with a curvature angle of more than 45°. Every step of image processing was performed in a blind way and MRI images with spurious brain connections due to possible noise effects on the whole brain network were excluded for network construction and statistical analyses.

### Network construction

**Figure 5:**
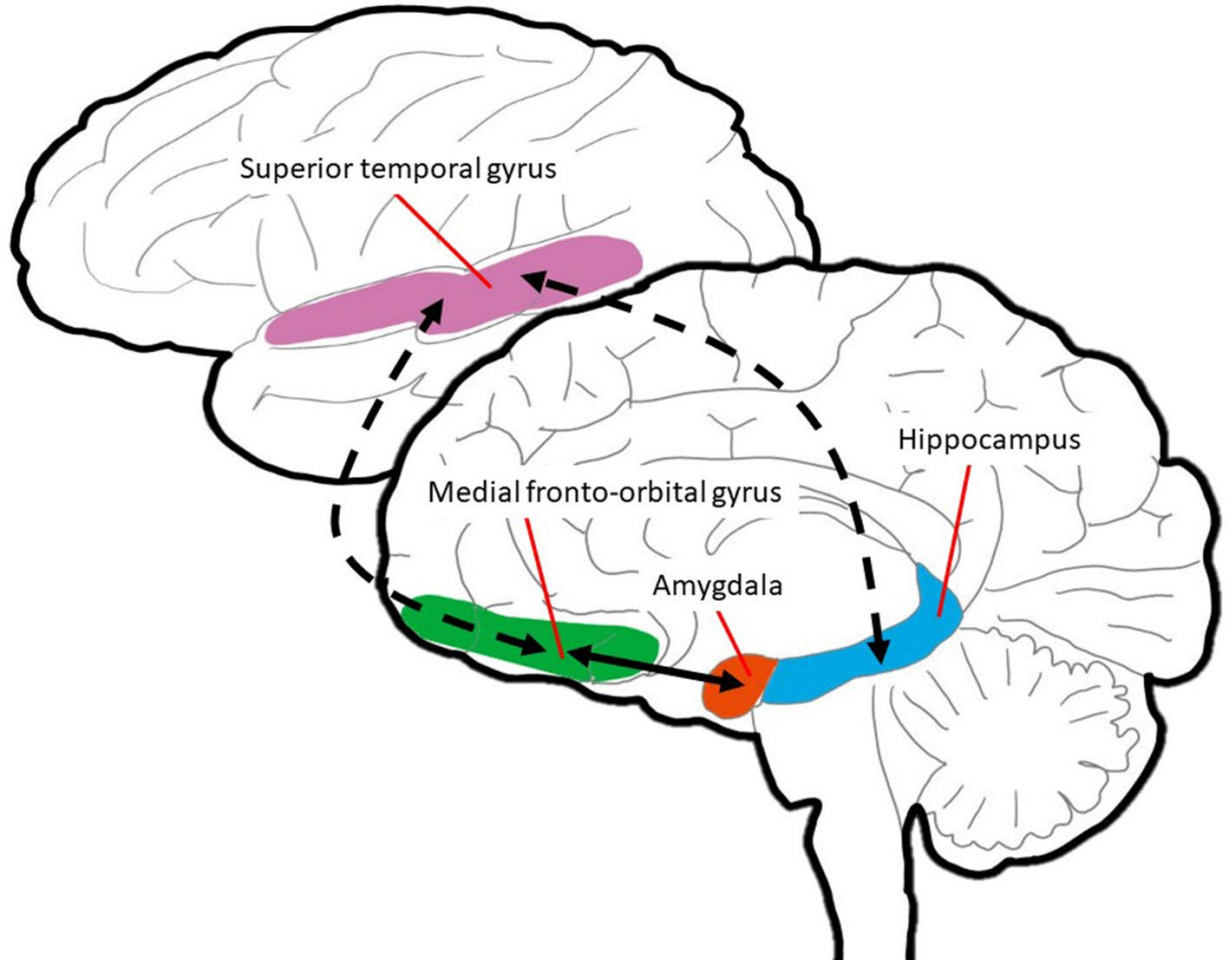
Schematic representation of fronto-temporo-limbic areas connected via medial and lateral cortical pathways. The brain diagram presented in the top register presents a lateral view, wherein the pink area indicates the superior temporal gyrus. The lower register presents a medial view of the medial fronto-orbital gyrus, amygdala, and hippocampus depicted in green, red, and blue, respectively. The circuit linking this structures is key to the mediation of responses to social–emotional stimuli by the prefrontal, temporal, and limbic systems.

Topological organizations of global structural networks were characterized with the Brain Connectivity Toolbox in MATLAB (The Mathworks, Inc., Natick, Massachusetts, USA)^26,27^. Network nodes were defined based on the Johns Hopkins University (JHU) neonate atlas. All neonatal FA images were nonlinearly transformed to the JHU neonate atlas, and labels in the JHU atlas space were inversely transformed to FA images using the inverse warping field. The 64 labels in the JHU neonate atlas were defined as nodes for an individual structure network. We used estimated fiber tracts in the whole brain for edge definition and quantified connectivity between cortical regions. The connectivity strength was then computed by obtaining the average FA value along the fiber tracts^4^. The average FA value was calculated by the arithmetic average of FA taken over all voxels comprising each fiber and then averaged over all fibers comprising the tract. Finally, we constructed unweighted 64 × 64 connectivity network matrices for each subject (Fig. 5).

### Network analysis

The global and local efficiency, characteristic path length, and small-worldness were computed to characterize the potential ease with which information could be transferred concurrently across a network and locally communicated neighborhood. The network integration refers to how efficiently and rapidly all the nodes integrate discrete specialized information. The most popular network integration measures include characteristic path length (L_p_) and global efficiency (E_glob_): a shorter path length and increased global efficiency suggest the potential integration of effective connections. The characteristic path length was defined by the average shortest path length in a network^28^.

$${L}_{p}=\frac{1}{N(N-1)}\sum_{i\in G}\sum_{j\in G,j\ne i}{L}_{ij}$$

where ${L}_{ij}$ is the shortest path length between nodes $i$ and $j$ and $N$ is the set of all nodes in the network $G$. The global efficiency was defined as the mean of the inverse shortest path length in a network^29.^

$$ E_{glob} = \frac{1}{{N\left( {N - 1} \right)}}\mathop \sum \limits_{i \in G} \mathop \sum \limits_{j \in G,j \ne i} {1 \mathord{\left/ {\vphantom {1 {L_{ij} }}} \right. \kern-\nulldelimiterspace} {L_{ij} }} $$

Segregation indicates the relative strength of within-network connections compared to integration. The most popular measures of segregation include the clustering coefficient and local efficiency. The clustering coefficient was defined as the ratio between the number of existing edges between neighbors and the number of all possible connections between neighbors^30^.

$${C}_{p}=\frac{1}{N}\sum_{i\in G}\frac{{2*E}_{i}}{{k}_{i}({k}_{i}-1)}$$

where ${k}_{i}$ is the degree of node $i$ and ${E}_{i}$ is the number of edges with the neighbors of node $i$.

The local efficiency of a node was calculated as the global efficiency of the neighborhood sub-network of this node. The local efficiencies across all nodes were averaged to estimate the local efficiency of the network^26^

$${E}_{local}=\frac{1}{N}\sum_{i\in G}{E}_{local, i}$$

where ${E}_{local, i}$ is the local efficiency of node $i$. Reflecting both integrated and segregated information processing, small-worldness was defined by the clustering coefficient and characteristic path length^28^.

$$\upsigma =\frac{{L}_{p}/{L}_{ran}}{{C}_{p}/{C}_{ran}}$$

where C_ran_ and L_ran_ are the mean clustering coefficient and characteristic path length of 1—random networks. All network metrics were calculated using the Brain Connectivity Toolbox (http://www.brain-connectivity-toolbox.net).

Informed by prior studies^9,11,31^, a regions-of-interest (ROI) connectivity analysis was performed to explore fronto-temporo-limbic regions, including the bilateral mFOG, STG, amygdala, and hippocampus (Fig. 5). The mFOG is a key cortical structure that plays an important role in social domains related to emotional regulation and behavioral suppression. The STG mediates the initial social perception of visual and auditory social cues in the temporal lobe. The amygdala and hippocampus are the limbic regions linked to social cognition and emotional processes through the fronto-limbic pathway. ROIs were defined based on the JHU neonate atlas. Betweenness centrality refers to how important a given node is to the efficient communication between the other nodes^32^. The betweenness centrality of a node was defined as the fraction of all shortest paths between any two nodes a and b that passes through a given node n. We characterized network lateralization of the betweenness centrality between the left and right structural networks of the medial fronto-orbital gyrus, STG, amygdala, and hippocampus. We thus represented fronto-limbic brain circuitry in terms of between centrality.

### Statistical analyses

SPSS 21.0 (SPSS, Chicago, IL) software was used for statistical calculations. We used Student’s t-test, Mann–Whitney U-test, Fisher’s exact test, and the chi-square analysis to compare clinical variables between groups. Based on the prevalences of impaired cognition or motor outcomes among very-low birth weight newborns (15% among term-born children and 40% among very preterm children)^33^, the required sample size to detect differences in prevalence between the study and general populations was 94 for all infants (52 for each group) with an alpha of 0.05, a desired power of 0.8, and a dropout rate 10%. For the term infants, the total sample size was smaller than the calculated sample due to lack of follow-up assessment. General linear models were generated to compare the network parameters and lateralization indices of the network parameters between preterm and full-term infants. The analysis of structural connectivity incorporated the global network matrix, including clustering, local efficiency, path length, global efficiency, and small-worldness, and the betweenness centrality of the fronto-limbic connections. A correlation analysis was performed to explore whether the age at MRI scan correlated with the lateralization index of the structural network. The subgroup analyses for the comparisons of structural network metrics between 57 preterm infants with abnormal Bayley-SE (< 1SD below average) and normal Bayley-SE (≥ 1SD below average) were performed using clinical variables such as gestational age and age at scan as covariates.

The asymmetry of the aforementioned network metrics was evaluated with the asymmetry score: AS = 100 × (X(L) − X(R))/(X(L) + X(R)), where X(R) and X(L) indicate the network parameters of the right and left hemispheres, respectively^17^. By incorporating the relative network metrics of both hemispheres into one value, the AS(X) index permits the assessment of differences between the right and left hemispheres. Ranging between + 100 and − 100, the AS(X) is positive when metric X shows prominent rightward asymmetry and negative when the opposite is the case. Lw measures the overall routing efficiency of the network and is inversely related to Eglobal. For a network, the higher the Lw, the less efficient the global integration. Hence, positive AS(Lw) indicates a leftward advantage of global integration, and negative AS(Lw) a rightward asymmetry of global integration. Continuous variables are represented as the mean ± SD. A *P* value of ≤ 0.05 was considered to indicate statistical significance. The resulting *P* values were corrected for the false discovery rate (FDR); FDR-adjusted *P* values of < 0.05 were considered to indicate statistical significance for multiple comparisons.
